# Echocardiographic Evaluation of Pulmonary Pressures and Right Ventricular Function after Pediatric Cardiac Surgery: A Simple Approach for the Intensivist

**DOI:** 10.3389/fped.2017.00184

**Published:** 2017-08-29

**Authors:** Maurice Beghetti

**Affiliations:** ^1^Pediatric Cardiology Unit, University Children’s Hospital HUG, Pulmonary Hypertension Program HUG, Centre Universitaire Romand de Cardiologie et Chirurgie Cardiaque Pédiatrique (CURCCCP), University of Geneva, Geneva, Switzerland; ^2^Pediatric Cardiology Unit, University Children’s Hospital HUG, Pulmonary Hypertension Program HUG, Centre Universitaire Romand de Cardiologie et Chirurgie Cardiaque Pédiatrique (CURCCCP), University of Lausanne, Lausanne, Switzerland

**Keywords:** hypertension pulmonary, echocardiography, pulmonary pressure, heart defect, congenital, postoperative period

## Abstract

Pulmonary hypertension (PH) is diagnosed using cardiac catheterization and is defined as an elevation of mean pulmonary artery pressure (PAP) greater than 25 mmHg. Although invasive hemodynamics remains the gold standard and is mandatory for disease confirmation, transthoracic echocardiography (TTE) is an extremely useful non-invasive and widely available tool that allows for screening and follow-up, in particular, in the acute setting. TTE may be a valuable alternative, allowing for direct measurement and/or indirect assessment of PAP. Because of the complex geometric shape and pattern of contraction of the right ventricle (RV), as well as the inherent complexity of cardiac repair, no single view or measurement can provide definite information on RV function and PAP and/or pulmonary vascular resistance. In addition, specific training and expertise may be necessary to obtain the views and measurements required. Some simple measurements may be of help when rapid evaluation is mandatory and potentially life saving: the assessment of tricuspid and/or pulmonary valve regurgitant jet and the use of the Bernoulli equation allow for measurement of PAP. Measurements such as the analysis of the pulmonary Doppler wave flow, the septal curvature, or the eccentricity index, assessing ventricular interdependence, are useful for indirect assessment. A four-chamber view of the RV gives information on its size, hypertrophy, function (fractional area change), and tricuspid annular plane systolic excursion as an evaluation of the longitudinal function. Based on these simple measurements, TTE can provide detection of PH, measurement or estimation of PAP, and assessment of cardiac function. TTE is also of importance in follow up of PH as well as providing an assessment of therapeutic strategies in the postoperative setting of cardiac surgery. However, PAP may be misleading as it is dependent on cardiac output and requires accurate measurements. In the presence of residual lesions, analyses of some Doppler measurements may be misleading and not reflect real PAP. Should the TTE evaluation reveal non-conclusive, invasive hemodynamics remains the gold standard.

## Introduction

Despite major advances in the understanding of the regulation of the pulmonary circulation, and in spite of the improvement in surgical results and the discovery of potential therapies targeting the pulmonary vascular bed in the pre- and postoperative period, pulmonary hypertension (PH) still remains a major problem in congenital heart disease after surgery (1).

Acute or chronic PH may punctuate the postoperative course despite accurate surgery. The primary therapeutic goal is to decrease pulmonary vascular resistance (PVR) and pressure and, if not possible, avoid stimulation of the pulmonary circulation; but the most important goal is to maintain right ventricular function and to support cardiac output through the balance of pulmonary to systemic vascular resistance (1).

However, there is no clear value of pulmonary artery pressure (PAP) or PVR that indicates a need for therapy in the postoperative period. A consensus would indicate that a mean PAP >25 mmHg or >60% the systemic pressure associated with signs of low cardiac output would indicate the need of specific therapy.

It is still difficult to predict the patients who would present with PH in the immediate postoperative period but patients older at time of repair of a left-to-right shunt with syndromes (i.e., trisomy 21) and elevated post capillary pressures (i.e., anomalous pulmonary venous return or neonatal mitral valve problems) seem to be at increased risk.

Because of the difficulty to predict which patient may develop symptomatic PH it also difficult to decide which patient may benefit from a pulmonary arterial catheter for monitoring PAP. Placement of pulmonary artery catheters should be minimized, and moreover the use of PA catheters may focus the attention of physician on PAP and delay progress. This underscores the need of non-invasive techniques to assess the PAP and right ventricular function such as transthoracic echocardiography (TTE). Several recent reviews describe the use and importance of TTE in the diagnosis and follow up of pediatric PH, but they mostly focus on chronic PH and are not specifically dedicated to the specific postoperative situation (2–5). Kaestner et al., in their review of pediatric PH in the intensive care unit, summarize some of the echocardiography measurements that are useful in this setting (6). In addition, they describe all potential techniques that may, in some cases, require experienced sonographers.

Transthoracic echocardiography is essential in congenital cardiology and particularly in pediatric cardiology. Cardiac anatomy can almost always be clearly defined in experienced hands. Measurements of right and left atria and ventricles’ sizes can be assessed as well as systolic and diastolic function. However, it must be kept in mind that they should be assessed in relation to the cardiac malformation. Doppler interrogation of all valves or shunts can be performed and pulmonary arterial pressures measured using the Bernoulli equation, or estimated *via* different modalities that we will discuss hereafter (Table 1). In this review, we will mainly describe simple TTE measurements that a neonatologist/pediatric intensivist can use keeping in mind that even a trained pediatric cardiologist may experience significant problems in this acute setting.

**Table 1:** Detection of elevated pulmonary arterial pressure.

Direct measurements	Indirect measurements
Tricuspid regurgitant jet (systolic pressure)	Septal curvature
Pulmonary insufficiency jet (diastolic and mean pressure)	Right ventricle (RV) ejection time
Post-tricuspid shunt gradient (systolic pressure)	RV acceleration time
	RV pre-ejection time
	Pulmonary Doppler flow morphology

Before presenting the potential measurements, it is important to remember some pathophysiological and anatomical concepts that are mandatory to analyze TTE imaging and measurements. PH is diagnosed using cardiac catheterization and is defined as an elevation of mean PAP greater than 25 mmHg (7). Although invasive hemodynamics remains the gold standard and mandatory for disease confirmation, echocardiography is a very useful non-invasive and widely available tool that allows for rapid screening and follow-up of PH and cardiac function. However, because of the complex geometric shape (inflow, trabecular portion, and infundibulum) and pattern of contraction (peristaltic contraction directed from inflow to infundibulum) of the right ventricle (RV), as well as the inherent complexity of cardiac repair, no single view or measurement can provide definite information on RV function and PAP and/or PVR.

It is also of importance to discuss the potential hemodynamic reasons that lead to increase in PAP. In patients with CHD and in particular with left-to-right shunts, the increase in mean PAP may be due to an increase in pulmonary blood flow and/or an increase in PVR or an increase in post capillary pressure. PVR cannot be directly and easily measured and is defined as the ratio of the mean fall of pressure across the pulmonary vascular bed divided by the pulmonary blood flow. PVR is thus derived from the formula mean PAP − LA/Q, where LA is the left atrial pressure, and Q is the pulmonary blood flow. From this formula, it appears clearly that an increase in PAP may either be due to an increase in pulmonary blood flow (i.e., a left-to-right shunt), an increase in PVR, or an increase in pulmonary venous pressure. An increase in post capillary pressure should be suspected in the presence of dilated and poorly functioning left ventricle, a severe mitral stenosis or regurge, or a dilated left atrium. Residual right ventricular outflow tract, pulmonary arterial or pulmonary venous obstruction must be excluded after surgical repair. This needs to be kept in mind. In addition, in the postoperative period, access to high-quality pictures and tracings are not always possible and extrapolations have to be made.

### Direct Measurement of Pulmonary Pressure

**Figure 1:**
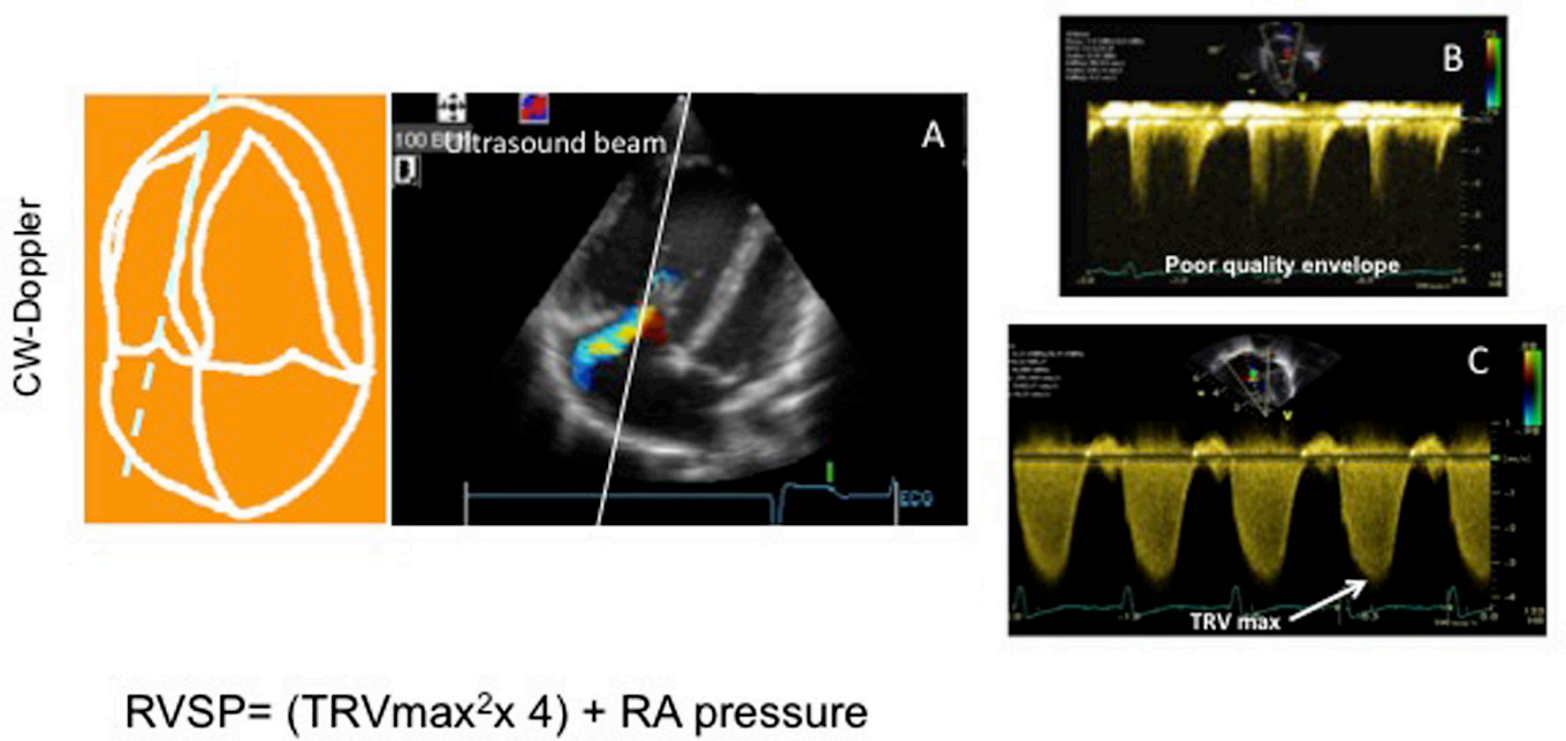
Tricuspid regurgitant velocity in a patient with pulmonary hypertension. **(A)** Four-chamber view showing a tricuspid jet the aligned ultrasound beam for assessment of tricuspid velocity. **(B)** Image showing a poor quality envelop of the tricuspid jet that does not allow accurate measurement. **(C)** Good tricuspid envelope allowing measurement of the maximal tricuspid regurgitant jet velocity (TRV_max_).

The easiest way of measuring systolic PAP is through the evaluation of the continuous wave flow Doppler of the tricuspid regurgitant (TR) jet, if present, and using the Bernoulli equation (Figure 1). The TR jet is usually assessed in the apical four-chamber view but can also be measured in the short axis view. The TR jet allows the measurement of right ventricular pressure: RVSP = 4 (TR peak velocity)^2^ + right atrial pressure. As for all Doppler evaluations, an accurate alignment of the beam of the ultrasound as well a complete envelope image is mandatory if accurate measurement is to be obtained. The right atrial pressure can be estimated using the inferior vena cava evaluation during inspiration in the subcostal view. The inferior vena cava can be dilated in patients with high PAP and RV dysfunction. It is measured in the subcostal view with the inferior vena cava entering the right atrium. Right atria pressure is estimated through the IVC diameter and the presence, or not, of inspiratory collapse. The IVC diameter of less than 2 cm that collapse >50% suggest a normal RA pressure of 3–5 mmHg, an IVC diameter >2 cm that collapse >50% suggest an RA pressure of >15 mmHg. It seems clear that, in children of different ages and size, these 2 cm are a problem and in small children the pressure may be evaluated on the percentage of the collapse during inspiration rather than as an absolute number (3). In addition, sometimes the subcostal view is not easy to obtain in the postoperative period. However, most of the patients in the immediate postoperative period have a central line with a central venous pressure reading that can be used for the calculation.

Once this RVSP value is obtained it must be remembered that the value of pressure is dependent of the cardiac output, and a low PAP or a decrease of PAP is not always a positive sign, if the cardiac output is low or has decreased. In addition, the RV systolic pressure reflects the PAP only in absence of any obstruction of the right ventricular outflow tract (pulmonary subvalvular, valvular, supravalvular obstruction, or pulmonary branches stenosis) (8).

**Figure 2:**
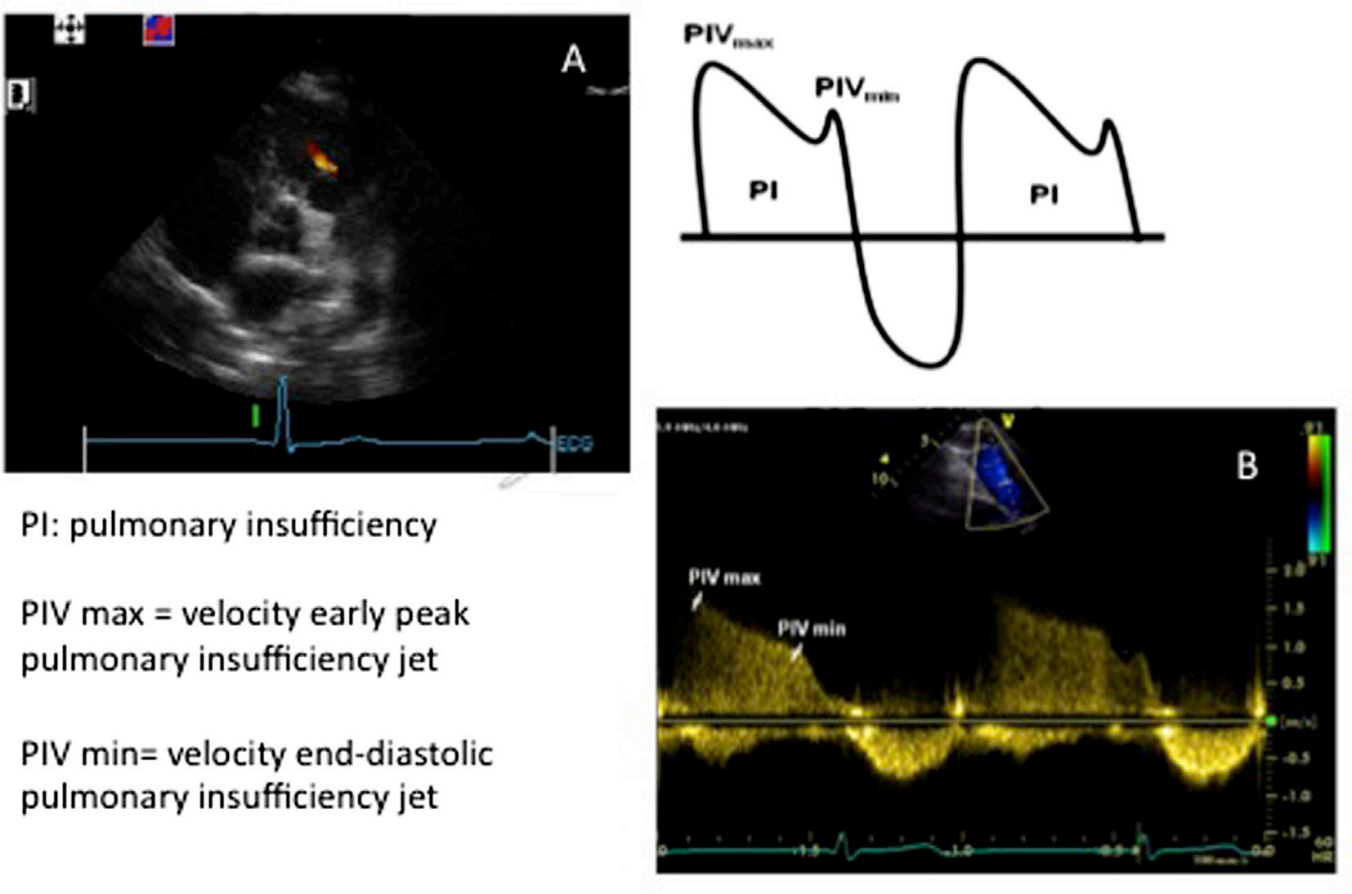
Images showing **(A)** short axis view and a pulmonary insufficiency jet and **(B)** Doppler envelope of the pulmonary insufficiency jet allowing for measurement of the peak velocity of the pulmonary insufficiency jet (PIV_max_) and the peak velocity of the end diastolic pulmonary insufficiency jet (PIV_min_). See the text for formulas.

Mean and diastolic PAP can also be estimated using the same Bernoulli equation through the pulmonary valve insufficiency jet Doppler assessment (Figure 2). The following equation allows for calculation.

$$\begin{array}{c} \text{Mean PAP}=4\;{\left(\text{velocity early peak pulmonary insufficiency jet}\right)}^{2}+\text{right atrial pressure}. \end{array}$$

$$\begin{array}{c} \text{Diastolic PAP}=4\;{\left(\text{velocity end diastolic pulmonary insufficiency jet}\right)}^{2}+\text{right atrial pressure}. \end{array}$$

The pulmonary insufficiency jet is usually obtained in the short axis view. As for the TR jet, high-quality tracing and alignment of the Doppler beam is a prerequisite for accurate measurements.

**Figure 3:**
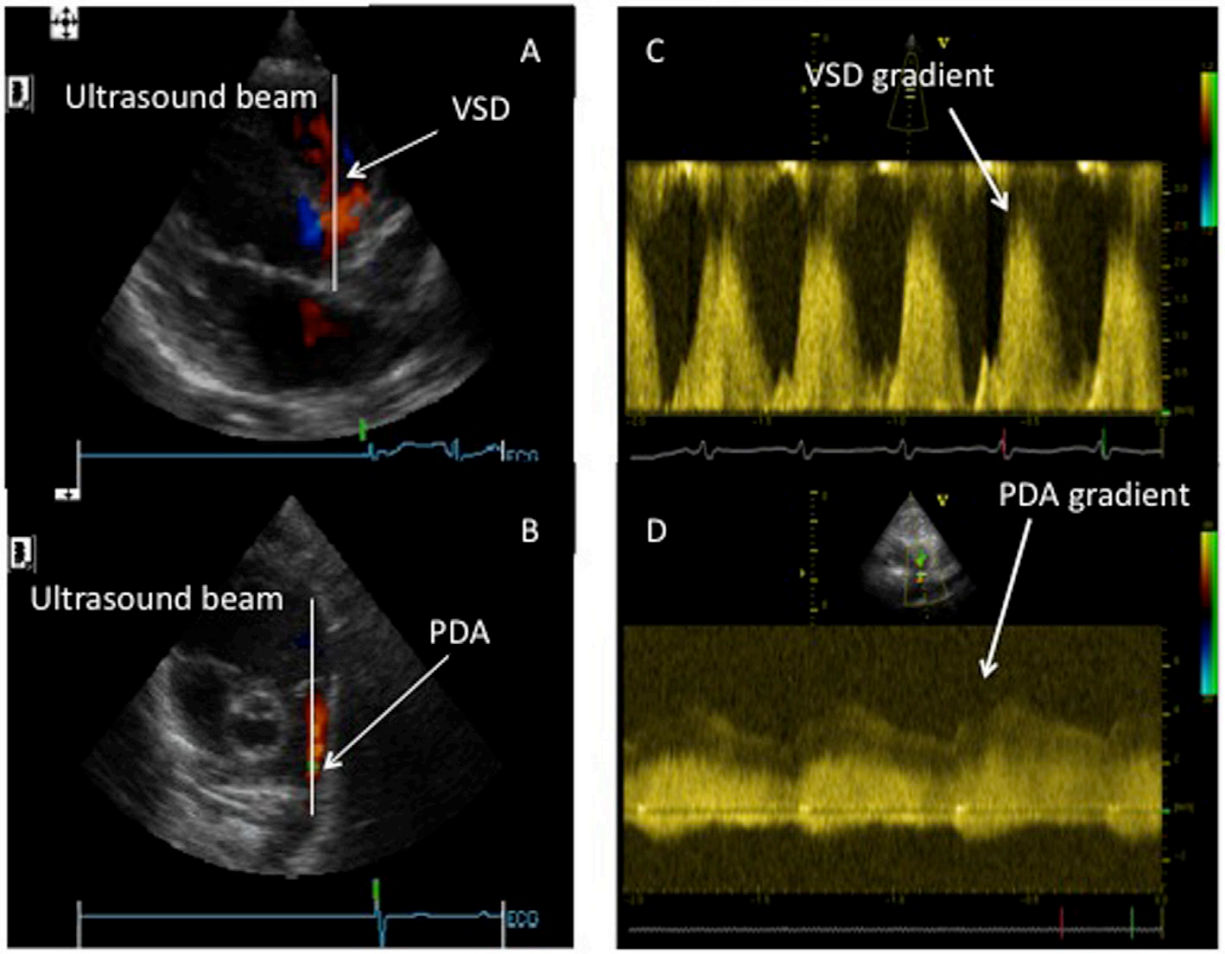
Assessment of systolic pulmonary pressure through the Doppler assessment of post-tricuspid shunts. **(A)** Doppler beam through a ventricular septal defect (VSD). **(B)** Doppler bean through a patent ductus arteriosus (PDA). **(C)** Doppler envelope of the VSD flow allowing for the measurement of the pressure gradient between systemic and pulmonary circulation. **(D)** Doppler envelope of the PDA flow allowing for the measurement of the pressure gradient between the systemic and pulmonary circulation.

In the presence of a post-tricuspid communication between the systemic and pulmonary circulation such as a ventricular septal defect or a ductus arteriosus, there is the possibility of measuring the systolic PAP using the velocity of the jet through these communications (Figure 3). The Doppler gradient reflects the difference of pressure between the systemic and pulmonary circulations. However, even more than for the TR jet, it is essential to align the ultrasound beam and obtain a perfect envelope for these Doppler assessments.

### Indirect Assessment of Pulmonary Pressure

If the PAP cannot be measured with the previously described Doppler assessments, either because there are no leaks through the pulmonary and tricuspid valves or because the Doppler envelopes are not of good enough quality, some other simple indirect assessments can be performed. In general, these measurements are more indicative of the presence, rather than the absolute measure of PH.

## Pulmonary Outflow Doppler Waveform

**Figure 4:**
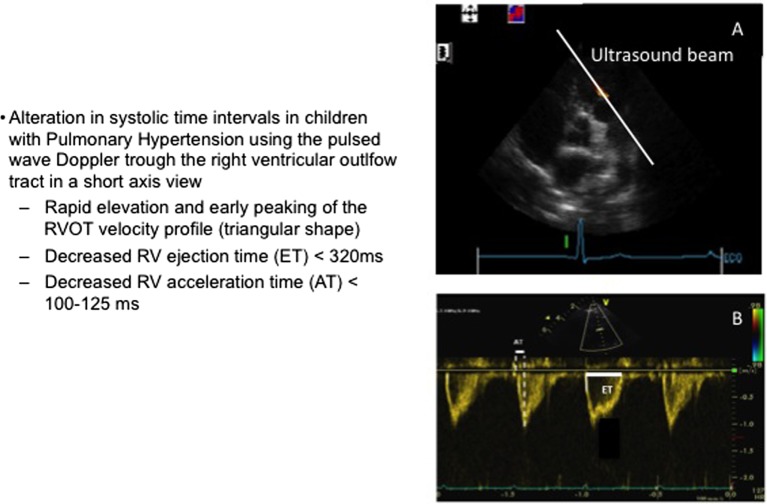
Right ventricular outflow tract Doppler evaluation. **(A)** Short axis view with the Doppler beam through the right ventricular outflow tract. **(B)** Pulmonary blood flow envelope. AT, acceleration time; ET, ejection time.

There are several measurements of the RV Doppler pattern that allow for evaluation of the PAP, but without giving a real value of this pressure (9–11). These measurements are described in Figure 4. Using the Doppler assessment through the right ventricular outflow tract, alterations in systolic time intervals can be observed such as delayed opening of the pulmonary valve, rapid elevation and early peak of the velocity (triangular shape of the wave form), decreased ejection time (ET <320 ms), decreased acceleration time (AT <100–125 ms), or increased pre-ejection time (PET >100 ms). It essential to remember that these values need to be adapted to age, body surface area, and sometimes heart rate (9–11). They all require good quality tracing to perform these measurements but can rapidly provide important information. However, there are very few data on their use in the acute setting after cardiac surgery, and most of these studies were performed in chronic PH.

## Eccentricity Index or Ventricular Interdependence

**Figure 5:**
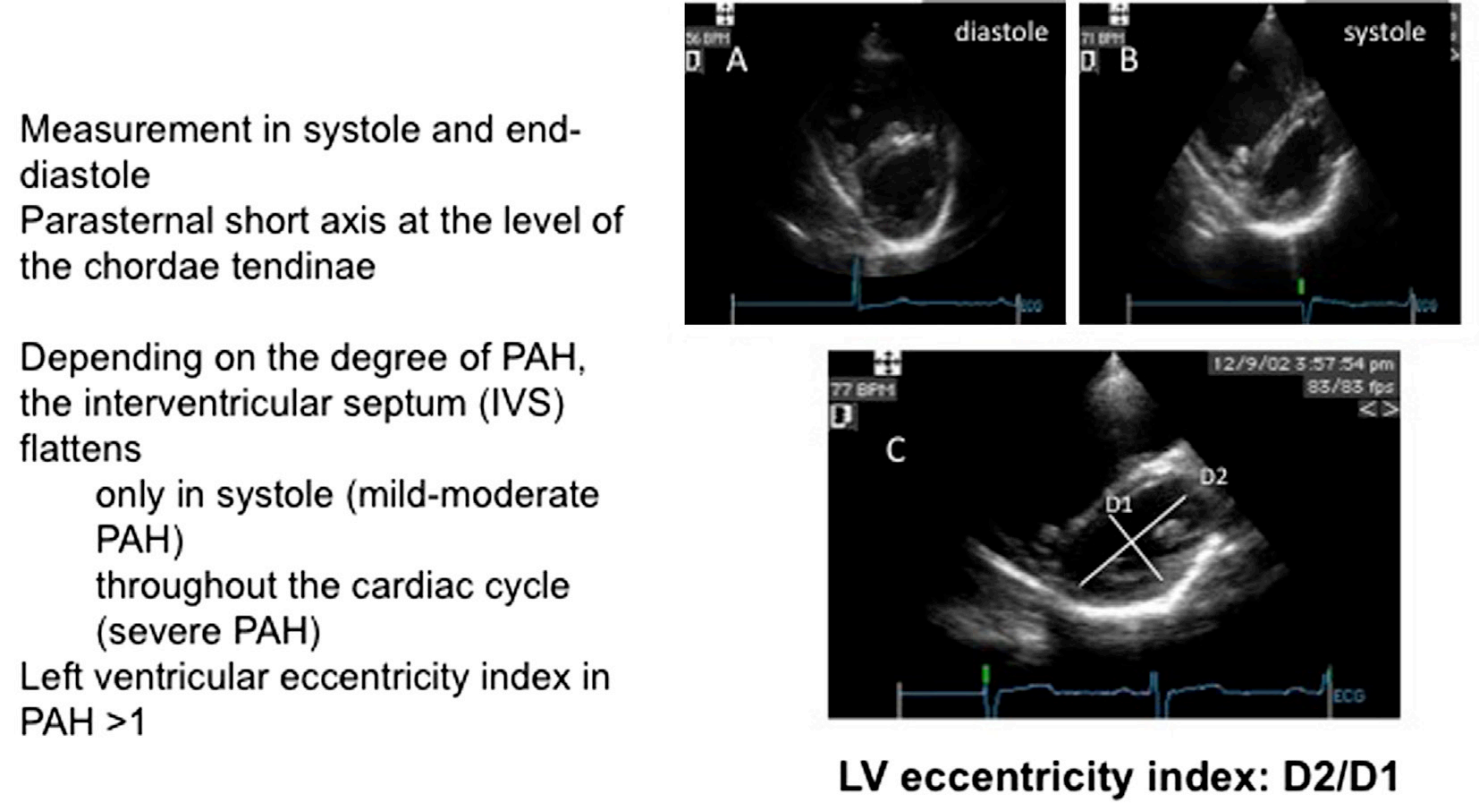
Assessment of the interventricular septal curvature. **(A)** Short axis view in diastole. **(B)** Short axis in systole. **(C)** Measurement of the LV eccentricity index.

As the heart is encircled by the pericardium, a change in the pressure or size of one of the ventricles will affect the other (12). This can be addressed using the short axis view by analyzing the ventricles size and in particular the anatomy of the interventricular septum (Figure 5). In normal conditions, when the systemic pressure is higher than the right, the septum bows to the right and you have a circular left ventricle. If the right ventricular pressure increases, and this in the absence of right outflow tract obstruction, the ventricular septum flattens during systole, creating the so-called D-shaped septum, or if the PAP becomes suprasystemic, it even bows to the left and compresses the left ventricle. Visual assessment of the septum as described above is enough to evaluate PAP, but the measurement of the systolic eccentricity index allows for a number that can be repeated to assess treatment efficacy. In normal conditions, the eccentricity index = 1. If this ratio exceeds 1, it is abnormal in favor of increased RV pressure (13).

This parameter can be considered as extremely useful in the intensive care setting as the importance of ventricular interaction is increasingly recognized, and it allows a simple assessment of ventricular interdependence.

### Pulmonary Vascular Resistance

In an ideal world, a measurement of the PVR would really reflect the severity of PH as it will include cardiac output (3). Several reports mention different formulas, all of them are quite complex, requiring several measurements (which increase the risk of mistakes). There are no reports of the regular and repeated use of these measurements in the acute setting, and they are usually reported in chronic patients. It seems reasonable to keep these calculations for experienced echocardiographists in specific settings as the formulas require multiple measurements and do not allow for rapid evaluation.

### Measurement of Right Ventricular Function

Evaluation of the RV is difficult, even for experienced sonographers. All the difficulties related to the RV function assessment by echocardiography have been described before, and the multiple reports using different methods attest for the difficulty to easily measure RV function by TTE, even more in the acute setting (3, 14). Some simple techniques will be described hereafter. Even if it would be considered as totally subjective, the visual “eyeball” assessment remains an evaluation that is still considered useful by many sonographers and gives a rapid indication of RV function.

## RV Fractional Area Change (RVFAC)

**Figure 6:**
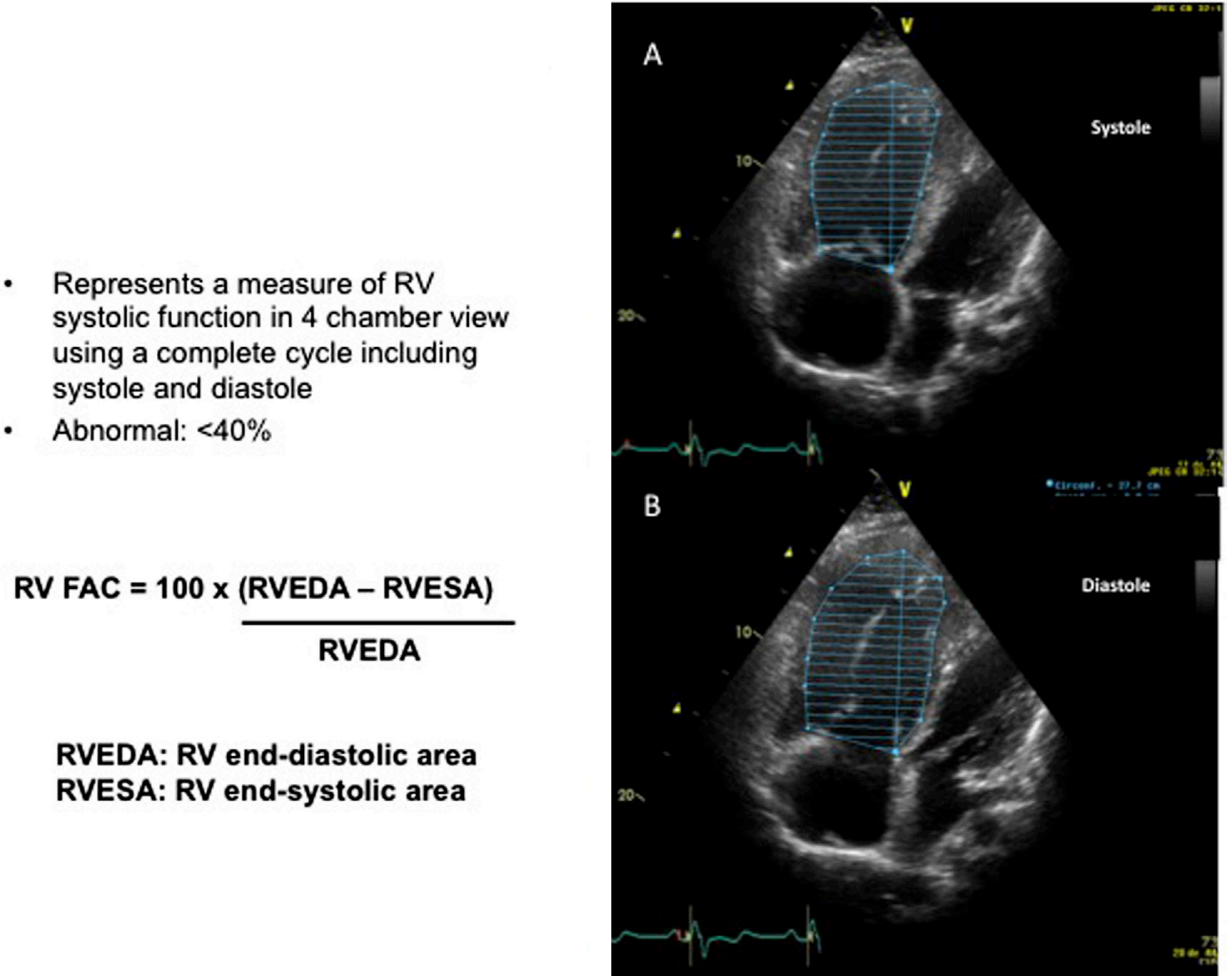
Measurement of the right ventricular fractional area change (RVFAC). **(A)** Four-chamber view allowing measurement of the surface of the right ventricle (RV) in systole (RVESA). **(B)** Four-chamber view allowing for the measurement of the surface of the RV in diastole (RVEDA).

The RVFAC measures the change of the surface of the RV between systole and diastole in the apical four-chamber view using the following equation: RVFAC = (end diastolic area − end systolic area/end diastolic area)/100. The major problem is to obtain an excellent apical four-chamber view, showing the complete RV as well as an accurate delineation of the endocardial border both in systole and diastole (Figure 6). Even if attractive, this is a quite a difficult assessment in the postoperative period.

## Tricuspid Annular Plane Systolic Excursion (TAPSE)

**Figure 7:**
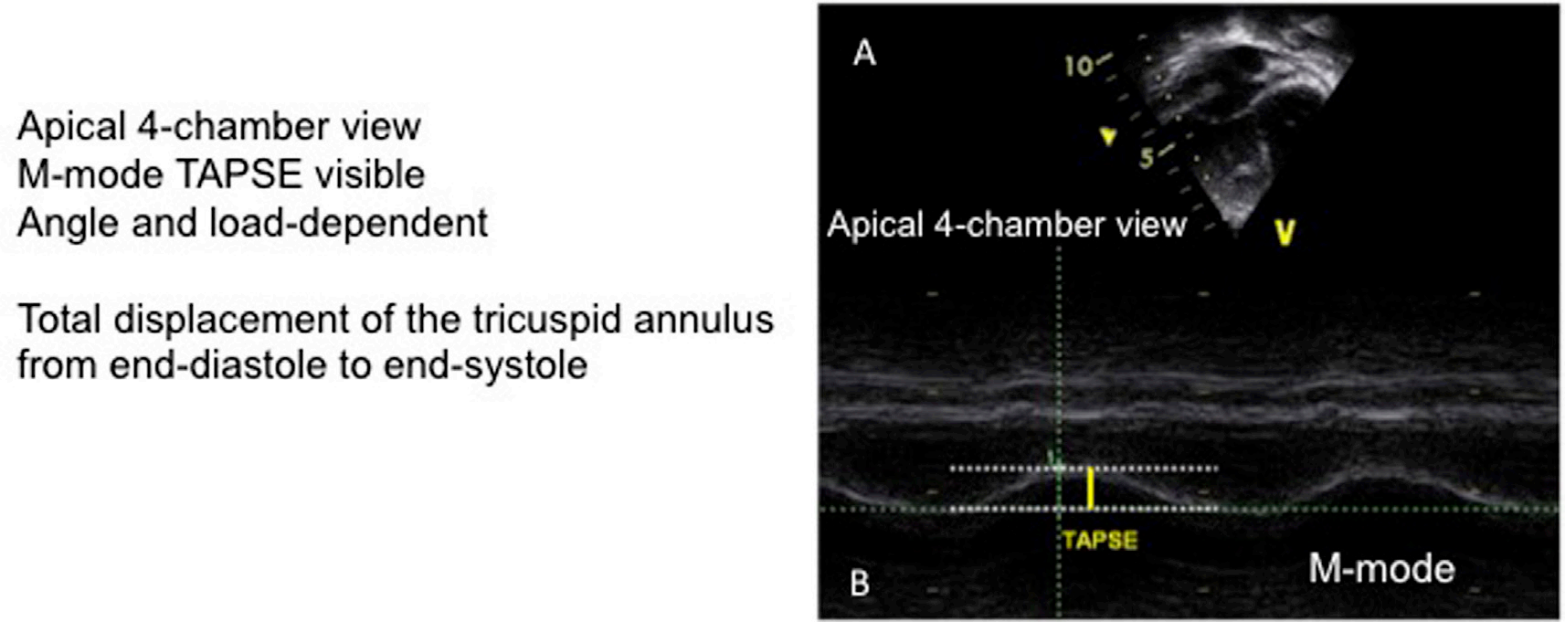
Tricuspid annular plane systolic excursion (TAPSE). **(A)** Four-chamber view of the right ventricle with ultrasound beam aligned on the tricuspid annulus. **(B)** Measurement of the TAPSE on the M mode image.

Tricuspid annular plane systolic excursion evaluates the systolic excursion of the tricuspid valve annulus in four-chamber view. The M mode cursor is placed through the lateral tricuspid annulus. As the RV function major contributor are the longitudinal fibers, it allows to measure the longitudinal function (Figure 7). This measurement has raised a lot of interest but mainly in the chronic follow up of PH (15–17). In adults, a value of <18 mm is considered abnormal. In children, this should be adapted to height and weight and *Z* score have been published (17). Reports in the acute setting are lacking. In addition, there are ongoing discussions about the validity of this measurement following cardiac surgery, in particular, if a patch has been placed in the septum or if a right ventriculotomy has been performed (18). Further studies on this simple parameter should be performed.

## Tissue Doppler Imaging

Even if a bit major complex, and requiring a specific software in the echo machine, this evolution seems attractive and useful. The scope of this manuscript is not to describe the technique of tissue Doppler imaging, but it measures the myocardial velocities in the apical four-chamber view, using the pulse wave Doppler placed at the level of the lateral tricuspid annulus, basal interventricular septum, and left ventricle lateral mitral annulus. Considering the RV, the s′ wave seems to be the most interesting measurement and correlates with RV function and also with PAP and PVR (19). Even if the data in the postoperative setting are not as conclusive as for chronic PH, it is worth considering an evaluation of these parameters in the future.

Multiple other modalities are available for the assessment of RV function but seems too complex, and not easy, to acquire for acute evaluation in the immediate postoperative evaluation and for non-expert sonographers. These are the TEI myocardial performance index, the strain and strain rate, and the three-dimensional assessment (3, 20). This may change in the near future with the improvement of the techniques. It is important to mention that, should the sonographer decide to obtain all the measurements, it can take a significant amount of time and may not be appropriate in an unstable patient, requiring rapid changes of treatment. This is the reason why this summary has been focused on simple, fast, and potentially, reproducible measurements, accessible to non-expert sonographers.

## Conclusion

In summary, echocardiography can provide detection of PH, estimation of PAP, and assessment of cardiac function both of the right and left ventricles. In the acute setting, and potentially unstable patients, it is important to have simple, rapid, accurate, and reproducible measurements. The TR jet, the mean and diastolic PAP septal morphology, as well as RV size and function through the use of RV fractional area and TAPSE are measurements that provide important information. As such, TTE is of importance in detection, follow-up, and assessment of therapeutic strategies in the postoperative setting of cardiac surgery. However, PAP may be misleading as it is dependent on cardiac output and requires accurate measurements. In the presence of residual lesions, analyses of some Doppler measurements may be misleading and not reflect real PAP. Should the TTE evaluation reveal not conclusive, invasive hemodynamics remains the gold standard!

## Author Contributions

MB is the only author of the manuscript.

## Conflict of Interest Statement

The author declares that the research was conducted in the absence of any commercial or financial relationships that could be construed as a potential conflict of interest. The handling editor declared a past co-authorship with the author MB.
